# Playing Mahjong for 12 Weeks Improved Executive Function in Elderly People With Mild Cognitive Impairment: A Study of Implications for TBI-Induced Cognitive Deficits

**DOI:** 10.3389/fneur.2020.00178

**Published:** 2020-03-27

**Authors:** Han Zhang, Yi Peng, Chunliu Li, Hong Lan, Guoqiang Xing, Zhu Chen, Bo Zhang

**Affiliations:** ^1^Department of Medical Rehabilitation, The Affiliated Hospital and the Second Clinical Medical College of North Sichuan Medical University, Nanchong Central Hospital, Nanchong, China; ^2^The Affiliated Hospital and the Second Clinical Medical College of North Sichuan Medical University, Nanchong, China

**Keywords:** mahjong, elderly, executive function, mild cognitive impairment, activities of daily living (ADL), TBI

## Abstract

**Background:** Mild cognitive impairment (MCI) is common among elderly people. So far, effective treatment that can stabilize or reverse the cognitive decline associated with MCI is lacking. Recent studies suggest that playing mahjong may improve attention and memory in elderly people. However, its effect on executive function remains unknown.

**Methods:** 56 elderly people (74.3 ± 4.3 years of age) with MCI from the First Social Welfare the First Nursing Home of Nanchong were randomized into mahjong and control groups (*N* = 28, each group). Subjects in the mahjong group played mahjong three times a week for 12 weeks, while people in the control group assumed normal daily activity. Executive function was evaluated using the Montreal Cognitive Assessment—Beijing (MoCA-B), the Shape Trail Test (STT), and the Functional Activities Questionnaire (FAQ) before the study and then at 6 and 12 weeks after mahjong administration.

**Results:** There were no baseline differences in MoCA-B, STT, and FAQ scoring between the two groups. The MoCA-B, STT, and FAQ scores, however, improved significantly in the mahjong group but not in the control group after the 12-week mahjong administration. Significant correlations were also found between STT and FAQ scores.

**Conclusions:** Playing Mahjong for 12 weeks improved the executive function of elderly people with MCI. Because Mahjong is a simple, low-cost entertainment activity, it could be widely applied to slow down or reverse the progression of cognitive decline in people with MCI, including those with traumatic brain injury.

## Introduction

Mild cognitive impairment (MCI) is a critical transient period of cognitive decline between normal aging and early dementia. Between 10 and 20 percent of adults above the age of 65 are diagnosed with MCI, and approximately 10 percent of MCI adults progress to Alzheimer's disease annually (1). The decline in executive function in elderly people with MCI is particularly obvious. The activity and executive function of the prefrontal cortex is significantly reduced with aging (2). Executive function is the advanced cognitive function for completing tasks and/or overcoming difficulties involving prefrontal cortex-mediated working memory and reflection, planning, organization, and management (2). The decline in executive function may have a negative effect on the instrumental activities of daily living (IADL). Marshall et al. as well as others, have shown a significant correlation between executive function and the ability to complete IADL in elderly with MCI (3). Mansbach et al. reported that most elderly with MCI were deficient in their abilities to perform IADL (4). Executive function was a stronger predictor for forecasting IADL dependence than memory, particularly for the performance of complex finances, the performance of complex cooking, and the ability to remember events (4). Executive function was more closely related to instrumental and advanced ADL than to basic ADL (5). Another study has shown that intervention to stabilize/improve executive function in the early stages of dementia may delay the decline of IADL and improve the quality of life of elderly people (6). Thus, there is a need to explore effective interventions that can improve executive function in elderly people.

Recent studies show that intellectual activities and hobbies involving mental workload can have therapeutic effects on cognitive function in elderly people (7). Cheng et al. reported progressive improvement in cognitive performance after patients who were affected by an early stage of dementia played mahjong (8). Playing mahjong has also been found effective in improving short-term memory, attention, and logical thinking in both middle-aged and elderly people (9). Improved episodic memory after playing mahjong may involve the activation of selective and divided attention, inhibition of interfering stimuli, and mobilization of manipulation skills (10). An enriched environmental, emotional stimulation, and interpersonal interactions during mahjong activity may also play a role in the reactivation of neural circuits in an aged brain.

Mahjong is a popular form of social entertainment for elderly people in China. It has win-or-lose gambling-like characteristics and is played among four players. In order to win, the participants need to focus and coordinate visual, mental, and manual activities. These repeated activities may improve executive function in aging.

Playing mahjong requires four players sitting around a square table with raised edges. A mahjong set includes 136–152 tiles (depending on the version of the game) and three dice. Players mix the tiles (called washing) and place the tiles face-down, building them into blocks two layers high. They take turns throwing the dice and receiving tiles according to the number thrown, as well as sorting and arranging their own tiles into some desired spatial sequence. They then take turns drawing one tile from the face-down pile and discarding a piece. It is important to memorize the tiles played and to predict other players' moves and use this information to build a strategy to maximize one's chances of winning. In completing sets, combinations of three tiles can be formed as either three of a kind (a pong) (one variation is four of a kind) or a sequence of three numbers in a row of one suit (a chee). During play, every player has to pay attention to certain tiles when they become available to form a pong (win the game). The player may call “pong” any time, even when it is not his/her turn if he/she has two of a kind and the latest discard gives him/her a pong. The player calling “pong” takes the discard and reveals all three pieces (11) (https://corp.mahjongclub.com/basic-rules; https://www.thesprucecrafts.com/how-to-play-mahjong-411827; https://www.youtube.com/watch?v=7WygnpfFbMQ; https://www.youtube.com/watch?v=tRCb_LOkEmQ).

So far, however, no study has directly examined the effects of mahjong play on executive function in elderly people with MCI. In this study, we examined executive function and IADL in older adults with MCI who played mahjong for 12 weeks.

## Methods

### Subjects and the Nursing Home

People 65 years of age or older were recruited from the First Social Welfare Nursing Home of Nanchong City, Sichuan Province, China. The nursing home was selected for this study because (1) the lifestyle of elderly people in the nursing home was relatively simple and homogeneous; 2) the nursing home always had enough participants to play mahjong games at a given time.

#### Inclusion Criteria Included

- 65 years of age or older;
- Diagnosis and confirmation of mild cognitive impairment, including reports of cognitive decline by self or nurses; assessment with the Beijing version of the Montreal Cognitive Assessment (**MoCA-B**), (cut-off range: 17–23 [illiterate], 20–24 [elementary school], 20–25 [middle- to high school and above]); Clinical Dementia Rating (CDR), 0.5–1.0;
- Prior experience and knowledge of how to play mahjong, but had not played mahjong in the past 6 months;
- Free of a handicap and/or disability that could interfere with mahjong playing.

#### Exclusion Criteria Included

- Inability to participate in mahjong due to disability (e.g., visual impairment, hearing impairment, other severe diseases);
- A psychiatric history;
- A neurologic disorder that could affect cognitive function;
- Inability to comply with the time frame of this study;
- Suffering from major depression diagnosed with Geriatric Depression Scale-15 (GDS-15).

People with depression were excluded because depression is an independent negative variable affecting IADL ability and neuropsychological functioning including executive control and episodic memory in people with MCI (12). Depressive symptoms are associated with cognitive decline and aging (13).

GDS-15 has a set of 15 depression-related questions for evaluating depression in elderly people. A Chinese version of this scale has good reliability and validity in assessing elderly people (14, 15). In this study, GDS-15 scores of 8 or above indicate a possible depression status, and people who scored 8 or above were therefore excluded from the study.

The Clinical Dementia Rating (CDR) scale was applied to measure the grade of MCI and to exclude with CDR-2 or CDR-3 in elderly (16).

The sample size calculation was performed based on data (effect size is 0.7, αis 0.05, power is 0.8)from literature using the statistical program G^*^Power3.1. The sample of each group (mahjong or control) comprised at least 34 subjects, with a total of 68 subjects needed to achieve statistical representativeness. 69 participants who fulfilled the eligibility criteria were randomized into a mahjong group (35) and control group (34), with a total of 56 subjects completing the whole experiment.

All study procedures were conducted in accordance with the Helsinki Declaration of 1975 and were approved by the Institutional Review Board of Nanchong Central Hospital. Written informed consent forms were obtained from all participants prior to the start of the study, and no monetary reward was offered to participants of the study.

## Experimental procedure

The participants were randomized into a mahjong group or control group according to a computer-generated random number table with a 1:1 allocation ration. All participants were assessed over the course of three visits. During the first visit, details of the study were presented and written informed consent was obtained. Patient information sheets were also obtained and neuropsychological tests were performed. The final two assessment visits included neuropsychological tests, conducted during the sixth week and the twelfth week of the experiment.

## Intervention

The mahjong group was instructed to play mahjong three times a week for 1 h each time for 12 weeks based on previous reports (17, 18). Each mahjong game was played by a group of four players randomly assembled at the time. The project researchers were responsible for scheduling and managing the mahjong event. If one or more of the scheduled players could not show up for a game for any reason, another resident would serve as the temporary substitute. The control group includes those residents who did not participate in any form of mahjong activity during the trial period except their normal daily activities. Participants were instructed to record their daily activities through a daily life sheet following the staff's instruction given to them 1 week before the study. Participants in the mahjong group played mahjong Monday, Wednesday, and Friday afternoons at two o'clock in the same location to increase their compliance. To control bias, all participants were asked to keep a record of regular daily activity on the daily life sheet under the supervision of the staff.

## Measures

The values of the Montreal Cognitive Assessment Scale—Beijing (**MoCA-B**) (19), the Shape Trail Test (STT) (20) and the Functional Activity Questionnaire (FAQ) (21) were assessed before and at 6 and 12 weeks after the start of the study by researchers who were not involved in the management of mahjong activity.

## The Montreal Cognitive Assessment—Beijing (MoCA-B)

MoCA, first developed by Nasreddine, a clinical research center for neurology at Charles LeMoyne Hospital in Canada, was used to screen elderly people with mild cognitive impairment (22). The scale includes eight cognitive domains with a best potential score of 30 points. Each correct answer accounts for 1 point, whereas a wrong answer or no answer accounts for 0 points. **MoCA-B** was translated and revised by Wen in 2008 to include some cultural modifications (23). **MoCA-B** is one of the most widely used MoCA versions in China with high sensitivity (83.8%) and specificity (82.5%).

## The Shape Trail Test (STT)

The STT is a series of Arabic numerals, combined with Chinese culture (20, 24) and consists of two parts. Part A consists of 25 digits. The subject is asked to connect the numbers as pre-instructed. Part B also contains 25 digits, but each digit appears twice, in both a circle and a square, and need to be alternately connected by the subjects as pre-instructed. That test also shows the acceptable level of area under the curve (AUC = 0.835), the sensitivity (84.6%), and specificity (66.7%) of STT in elderly people (> 65 years) with an education level <12 years.

## The Functional Activities Questionnaire (FAQ)

The Functional Activities Questionnaire (FAQ), as a self-reported questionnaire, is used to differentiate the independence of instrumental activities of daily living (IADL). The FAQ is composed of ten items including simple finances, complex finances, shopping, games, simple cooking, complex cooking, current events, tracking media, remembering events, and transportation; the ability to perform each activity is rated from 0 (normal) to 3 (dependent) with a score range of 0–30. The higher the score, the more serious the damage of IADL function.

### Statistical Analysis

Data were analyzed using SPSS 22.0 software. The baseline differences and the differences after 6 and 12 weeks of mahjong intervention in **MoCA-B**, STT, and FAQ scoring between the two groups were analyzed using independent *t*-test and/or repeated measures analysis of variance as appropriate. A paired *t*-test was used to analyze the before-and-after treatment differences within each treatment group. *P* < 0.05 was considered statistically significant.

## Results

**Figure 1:**
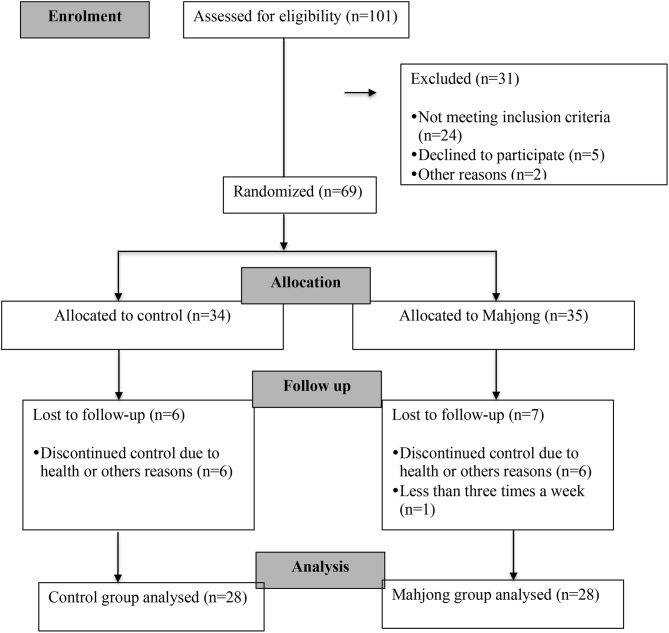
Flowchart of selection of subjects.

A total of 69 elderly people were recruited to participate in this study. The subjects were randomly assigned to the mahjong intervention group (*n* = 35) or to the control group (*n* = 34). During the intervention period, seven individuals from the mahjong group and six from the control group were excluded, leaving 28 in the mahjong group and 28 in the control group. The process for the selection of subjects is described in Figure 1.

The average age of all participants was 74.3 ± 4.3; the male-to-female ratio was 15:41 (26.8 to 73.2%), the education time was 5.7 ± 3.9 years, and the proportion of illiteracy (level of or under junior high school, and senior high school) was 21.4, 64.2, and 14.3%, respectively. The values in our present study are consistent with the recent report of elderly Chinese patients in another study by Jiang, 2008. The mahjong group did not differ significantly from the control group in age (74.4 ± 3.9 vs. 74.2 ± 4.8 years) (*p* > 0.05), years of education (5.6 ± 3.7 vs. 5.9 ± 4.2) (*p* > 0.05), proportion of female (78.6, vs. 67.9%) (*P* > 0.05), or CDR (0.88 ± 0.59 vs. 0.84 ± 0.62) (*p* > 0.05) (Table 1).

**Table 1:** Demographics of the participants (*N* = 56).

	**Mahjong**	**Control**	**Total**	***P***
Gender, (women %)	78.6%	67.9%	73.2%	0.375
Age, year (mean ± s.d)	74.4 ± 3.9	74.2 ± 4.8	74.3 ± 4.3	0.879
Education, year (mean ± s.d)	5.6 ± 3.7	5.9 ± 4.2	5.7 ± 3.9	0.814
GDS (mean ± s.d)	3 ± 1.56	2.86 ± 1.7	2.93 ± 1.64	0.748
CDR (mean ± s.d)	0.88 ± 0.59	0.84 ± 0.62	0.86 ± 0.6	0.826

*GDS-15, Geriatric Depression Scale; CDR, The Clinical Dementia Rating*.

A trend level difference existed in the baseline Montreal Cognitive Assessment (**MoCA-B)** between the mahjong (21.11 ± 2.22) and control (22.18 ± 2.39) groups (*t*/*P*-values =-1.74/0.09). Trend level differences were also found after 6 weeks (21.3 ± 1.9 vs. 22.1 ± 2.3, *t*/*P*-values =-1.4/0.15) and 12 weeks (22.8 ± 1.7 vs. 22.0 ± 1.9, *t*/*P*-values =1.63/0.11) of mahjong playing. However, an opposite direction of change in **MoCA-B** occurred between the two groups, i.e., a small but steady increase in the mahjong group and a small but steady decrease in the control group after 6 and 12 weeks of mahjong playing. The control group analysis showed that the MoCA-B score was significantly improved in the mahjong group after 12 weeks of mahjong intervention (22.8 ± 1.7) compared to the baseline (21.11 ± 2.22) (*P* < 0.01) and 6-week values (21.3 ± 1.9) (*P* < 0.01). But no such change was found in the control group (Table 2).

**Table 2:** Changes in **MoCA-B**, STT-B, and FAQ scores (mean ± s.d) after 6 and 12 weeks of mahjong intervention.

	**Baseline**	**6 weeks after intervention**	**12 weeks after intervention**	**t/*P*-value** **base vs. 6 w**	**t/*P*-value** **base vs. 12 w**	**t/*P*-value** **6 w vs. 12w**
**MoCA-B**						
Mahjong	21.11 ± 2.22	21.3 ± 1.9	22.8 ± 1.7	−1.000/0.326	−6.971/0.001^**^	−6.162/0.001^**^
Control	22.18 ± 2.39	22.1 ± 2.3	22.0 ± 1.9	0.493/0.626	0.895/0.379	0.593/0.558
t/*P*-value	−1.74/ 0.09	−1.47/0.15	1.63/0.11			
**STT-B**						
Mahjong	573.1 ± 113.8	555.1 ± 115.0	535.7 ± 111.7	8.88/ <0.001^**^	8.59/0.001^**^	5.79/ 0.001^**^
Control	559.3 ± 95.9	561.4 ± 102.0	565.5 ± 93.0	−0.28/0.783	−1.71/0.66	−0.45/0.098
t/*P*-value	0.49/ 0.62	−0.22/0.83	−1.084/0.283			
**FAQ**						
Mahjong	17.89 ± 4.64	16.9 ± 4.5	15.6 ± 4.8	5.50/ <0.001^**^	5.44/ <0.001^**^	2.86/ <0.01^**^
Control	19.36 ± 3.81	19.5 ± 3.3	19.9 ± 3.6	−0.87/0.39	−1.89/0.07	−1.8/0.083
t/*P*-value	−1.291/ 0.20	−2.61/0.012^*^	−3.74/0.00^**^			

* P < 0.05, difference between mahjong and control groups or between different times after mahjong intervention within each group;

** *P < 0.001, difference between mahjong and control groups or between different times after mahjong intervention within each group; base, baseline; STT-B, the Shape Trail Test-B; FAQ, Functional Activities Questionnaire; MoCA-B, Montreal Cognitive Assessment—Beijing*.

Similarly, no significant difference was found in STT between the mahjong and control groups at the baseline (573.1 ± 113.8 vs. 559.3 ± 95.9, *P* > 0.05), 6 weeks after mahjong intervention (555.1 ± 115.0 vs. 561.4 ± 102.0, *P* > 0.05), and 12 weeks after mahjong intervention (535.7 ± 111.7 vs. 565.5 ± 93.0, *P* > 0.05). Group time analysis showed continuously reduced STT scores in the mahjong group after 6 weeks (555.1 ± 115.0) (*P* < 0.01) and 12 weeks (535.7 ± 111.7) (*P* < 0.01) of mahjong playing compared to the baseline STT scores (573.1 ± 113.8). But no such reduction in STT scores was found in the control group (Table 2).

**Figure 2:**
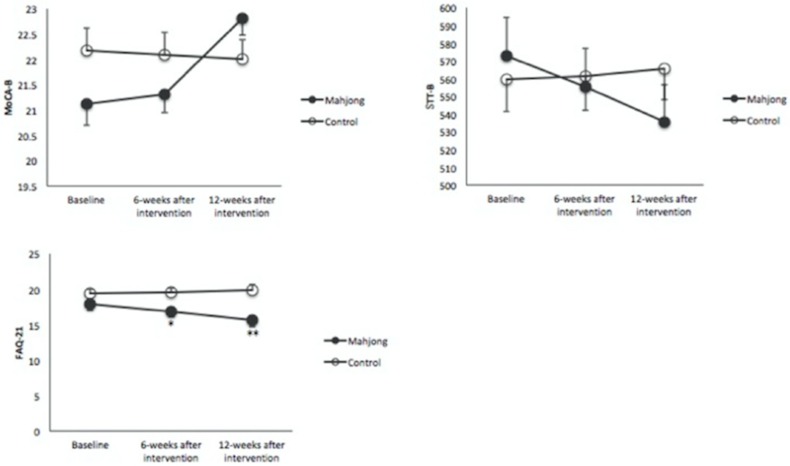
Changes in the Montreal Cognitive Assessment–Beijing (MoCA-B), the Shape Trail Test-B (STT-B), and Functional Activities Questionnaire-21 (FAQ21) among the studied groups. A significant effect of time is observed on the FAQ21 between the two groups after 6 and 12 weeks, while no significant changes were observed in MoCA-B and STT-B. *p < 0.05, **p < 0.001 significant between groups at point of assessment.

Compared to the baseline FAQ values (17.89 ± 4.64), the FAQ score decreased significantly and continuously in the mahjong group after 6 weeks (16.9 ± 4.5) (*P* < 0.01) and 12 weeks (15.6 ± 4.8) (*P* < 0.01) of mahjong intervention but not in the control group. The FAQ scores of the mahjong group became significantly lower in the mahjong group than in the control group after 6 weeks (16.9 ± 4.5 vs. 19.5 ± 3.3, *P* < 0.05) and 12 weeks (15.6 ± 4.8 vs. 19.9 ± 3.6, *P* < 0.01) of mahjong playing (Figure 2).

The relationships between the scores of the MoCA-B and STT and the scores of the FAQ are shown in Table 3. Significant correlations were found between MoCA-B and FAQ and between FAQ and STT.

**Table 3:** Correlations between FAQ, MoCA-B, and SST-B before and after 6 and 12 weeks of mahjong playing of all participants (*N* = 56).

	**FAQ:** **baseline**	**FAQ:** **6 weeks**	**FAQ:** **12 weeks**	**MoCA-B:** **baseline**	**MoCA-B:** **6 weeks**	**MoCA-B:** **12 weeks**	**STT-B** **baseline**	**STT-B** **6-week**	**STT-B** **12-week**
FAQ: baseline	1								
FAQ: 6 weeks	0.956^**^	1							
FAQ: 12 weeks	0.872^**^	0.923^**^	1						
MoCA-B: baseline	−0.583^**^	−0.480^**^	−0.444^**^	1					
MoCA-B: 6 weeks	−0.563^**^	−0.465^**^	−0.456^**^	0.931^**^	1				
MoCA-B: 12 weeks	−0.495^**^	−0.490^**^	−0.569^**^	0.766^**^	0.759^**^	1			
STT-B: baseline	0.681^**^	0.603^**^	0.566^**^	−0.494^**^	−0.483^**^	−0.362^**^	1		
STT-B: 6 weeks	0.703^**^	0.653^**^	0.615^**^	−0.471^**^	−0.444^**^	−0.364^**^	0.956^**^	1	
STT-B: 12 weeks	0.720^**^	0.676^**^	0.671^**^	−0.436^**^	−0.424^**^	−0.388^**^	0.957^**^	0.937^**^	1

** *Correlation is significant at the 0.01 level (two tailed)*.

*STT-B, the Shape Trail Test-B; FAQ, Functional Activities Questionnaire; MoCA-B, Montreal Cognitive Assessment—Beijingß*.

## Discussion

In this study, we evaluated the effects of mahjong playing on executive function in elderly people with MCI by using three scales, the MoCA-B, STT, and FAQ.

MoCA-B is a reliable screening tool for detecting different types of MCI, including amnestic MCI and non-amnestic MCI (25). According to a recent study of elderly Chinese participants with MCI, the cut-off values of MoCA-B for illiterate people (<1 year of education) with MCI is 17–23 points, for people with elementary school education, the boundary values are 20–24 points, and for people with junior high school education and above, the boundary values are 20–25 points (26).

The Trail Making Test (TMT) is one of the most sensitive and popular scales for testing executive function in people with MCI (27). In the test, subjects need to shift the focus of attention between various external stimuli when facing two cognitive tasks. The ability to shift tasks is a main component of executive function. Because TMT is based on the Latin alphabet, this limits its application in Chinese-speaking populations. STT is based on the Trail Making Test (TMT), which was developed for people who speak Chinese as a first language (20).

The Functional Activities Questionnaire (FAQ), as a self-reported questionnaire, is used to evaluate the independence of instrumental activities of daily living (IADL). Nitrini (28). reported that a FAQ score ≥6 indicates a functional impairment. FAQ is more sensitive in differentiating subjects with MCI from those without MCI when compared with other self-reported IADL scales (29).

Executive function consists of inhibition—responding appropriately to the needs of a task and/or specific objectives with controlled behaviors, thoughts, emotions and attention, and updates that allow for the retaining and manipulating of information to external tasks or stimuli—and cognitive flexibility, which allows one to modify one's behavioral response to external stimuli (30). Executive function plays an important role in completing complex activities like housekeeping, laundry, meal preparation, medication management, shopping, and transportation; the simultaneous activation of frontal cortex circuitry is involved in executive function (31).

It was first reported in 2006 that playing mahjong can significantly improve the cognitive function of patients with dementia (18). In a separate study, these authors also showed that mahjong playing had produced better outcomes than tai chi exercise in improving the cognition function of elderly people. Lu et al. reported that playing mahjong can improve short-term memory, attention, and logical thinking in elderly people (9). However, these studies have not examined the effects of mahjong on executive function and ability of life activity.

Our current study focused on the executive function and activity of daily living and showed that playing mahjong improved the executive function of elderly people with MCI. This finding may reflect the fact that playing mahjong is mentally and intellectually challenging. In order to win the game, participants need to concentrate as well as judge and predict the next moves of others in order to select the best strategies to win. Eye and hand movements are also required to play mahjong. All these activities may have mobilized cognitive reserves in the brain and thus enhanced executive function.

Recent studies suggest reserved neural plasticity in the structure and function of the prefrontal lobes of elderly people. Training elderly people to perform a series of cognitive tasks involving working memory and integrated cognition delayed the shrinkage of the prefrontal cortex and improved white matter integrity, functional connection, and the differentiation of neural networks (32–35).

The significantly improved cognitive function after 12 weeks of mahjong playing in elderly people with MCI is consistent with previous reports that playing mahjong has significant effects on memory, attention, and ability to think (36). Our results, however, differ from a 2014 report by Cheng et al. that shows no difference between the mahjong group and the control group after mahjong intervention (8).

This difference may reflect the fact that we used MoCA-B in our study, whereas Cheng et al. used MMSE in their study. It is known that MoCA-B is more sensitive than the MMSE in evaluating the cognitive function of elderly people in the Chinese population (37). MoCA-B covers a wider range of cognitive deficits, whereas MMSE is suitable for detecting memory and language impairment. The application of MMSE in evaluating executive and visual–spatial impairment is also limited. It has been reported that MoCA-B has a higher sensitivity (78%) than MMSE (67%) in detecting early MCI (22).

In this study, we observed subjects with MCI whereas Cheng's team studied subjects with dementia who have fewer cognitive reserves and are more resistant to treatment than those with MCI (38). Thus, subjects with MCI may have more cognitive reserves to effectively engage in mahjong activity and activate the cerebral cortex than subjects with dementia.

We also studied the relationship between executive function and instrumental daily living ability. The significant correlations between severe executive dysfunction and a worsening ability of daily living in this study are consistent with previous reports (3, 39). Activities of daily life include the critical cognitive ability of executive function tasks including complex problem solving, attention shifting between tasks, inhibiting irrelevant information, recalling lists of items, and sustaining attention on tasks (40).

Increasing evidence supports a correlation between MCI and a decline in everyday functioning, which includes basic and instrumental ADL, self-care tasks, and living independently in a community setting (5, 40). Correlations between apathy, depression, memory and executive functioning, dependence in IADL, and falling and hospital readmissions have also been reported, (12, 40).

Because executive function is projected in the prefrontal cortex, which is sensitive to aging-related brain atrophy and traumatic brain injury (TBI), it would be interesting to know if the present findings can be applied to prevent or reverse the decline in prefrontal cortex functional activity and the ability of daily living of TBI patients (41). It is tempting to speculate that playing mahjong could stimulate activity and restore some of the lost functions of prefrontal cortex and thus improve the executive function and instrumental activities of daily living in TBI patients because improved social interactions are important for restoring executive function and instrumental activities of daily living in subjects with TBI (42). When participating in multi-person games like mahjong, the participants are involved in social interactions and therefore executive functions may be synchronized or boosted among the participants. These social interactions may be critical for strengthening the neural network and improving physical and executive functions (43, 44). Findings suggest that the playing of mahjong can likely prevent loneliness and be beneficial for psychological well-being (45). Playing Mahjong can improve hand–eye coordination and manual dexterity, as evidenced by quickened speed of finger activity and shortened response time. A Japanese study confirmed that the flexibility of hand function affects executive function in elderly people and that improved physical function by means of playing mahjong is also reflected in the enhanced activity of the hand (46).

Although it is not clear yet whether the benefits of playing mahjong are due to regular human interaction with peers or due to playing mahjong itself, the extensive engagement of human interaction could be the most critical factor in producing the beneficial effect. It is known that loneliness and social isolation are major determinants of mental well-being, especially among older adults (47). One recent review suggests that narrative activity-based social interventions (as opposed to dancing, gardening, or other physical activities) can bring about positive well-being on social and health-related measures in older people living in nursing homes or similar institutions (48).

However, mahjong is a four-player gambling game, which originated in China and is played in Chinese communities across the world, so financial problems are particularly concerning for older adults or TBI patients, since they are likely to be on fixed incomes or not working (49). In addition, prolonged mahjong activity could increase the risk for serious health conditions like hypertension and heart disease because of exposure to both direct and secondhand smoke. Playing mahjong also often involves money, which can be a stressful activity, especially when financial losses, marital discord, or other social problems may increase the risk for chronic diseases like diabetes and hypertension (50). There are case reports that mahjong could cause reflex seizures that are possibly induced by non-verbal cognitive tasks (51).

This pilot study has limitations. The number of participants is small because of drop-out, and no long-term follow-ups were conducted. Although the self-reported FAQ was used to evaluate ADL, is the questionnaire is considered not as accurate as performance-based evaluations because it lacks sound psychometrical properties and its potential over- or underestimation of functional ability (4, 5). Future research could be done to determine the mechanisms of mahjong on executive function in elderly people with MCI and to ascertain the effects of long-term mahjong game on executive function in TBI patients.

## Conclusion and implications for TBI

This study showed that playing mahjong for 12 weeks could improve executive function and the ability of daily activity in elderly people with MCI. Considering that many people with TBI also suffer cognitive deficits and executive dysfunction, the present findings suggest that mahjong as a cognitive game may be a potential method to improve these cognitive deficits and the activities of daily living in people with TBI. The time of participation and health conditions need to be considered when the mahjong playing method is practiced in TBI patients because of possible disadvantages.

## Data Availability Statement

The raw data is not publicly available. However, the raw data could be available by the corresponding author upon reasonable request.

## Ethics Statement

All study procedures were conducted in accordance with the Helsinki Declaration of 1975 and were approved by the Medical Ethics Committee of Nanchong Central Hospital. Informed consent was obtained from all participants prior to enrollment into the study.

## Author Contributions

BZ, HZ, YP, and GX conceived the study. HZ, YP, CL, HL, and ZC contributed to the experimental implementation, participant enrollment, evaluation, follow-up, and data collection. HZ and GX contributed to the data analysis, literature search, and manuscript preparation. All authors have read and approved the manuscript.

### Conflict of Interest

The authors declare that the research was conducted in the absence of any commercial or financial relationships that could be construed as a potential conflict of interest.

## Abbreviations

ADL: activities of daily living
FAQ: functional activities questionnaire
GDS-15: geriatric Depression Scale 15-item version
IADL: instrumental activities of daily living
MCI: mild cognitive impairment
MoCA-B: montreal cognitive assessment (Beijing edition)
STT: Shape Trail Test
TMT: trail making test.

## References

[1] Carnicelli L Maestri M Di Coscio E Tognoni G Fabbrini M Schirru A. A longitudinal study of polysomnographic variables in patients with mild cognitive impairment converting to Alzheimer's disease. J Sleep Res. (2019) 28:e12821. 10.1111/jsr.12821 30724408

[2] Hamasaki A Akazawa N Yoshikawa T Myoenzono K Tagawa K Maeda S. Age-related declines in executive function cerebral oxygenation hemodynamics. Tohoku J Exp Med. (2018) 245:245–50. 10.1620/tjem.245.245 30101827

[3] Marshall GA Rentz GM Frey MT Locascio JJ Johnson KA Sperling RA Executive function and instrumental zctivities of daily living in MCI and AD. Alzheimers Dement. (2011) 7:300–8. 10.1016/j.jalz.2010.04.005 21575871 PMC3096844

[4] Mansbach RAM William E Differential contributions of memory and executive functions.pdf. Gerontologist. (2018) 10:1–11. 10.1093/geront/gny097 30137363

[5] Cornelis E Gorus E Van Schelvergem N De Vriendt P. The relationship between basic, instrumental, and advanced activities of daily living and executive functioning in geriatric patients with neurocognitive disorders. Int J Geriatr Psychiatry. (2019) 34:889–99. 10.1002/gps.5087 30761619

[6] Kirova AM Bays RB Lagalwar S Working memory and executive function decline across normal aging, mild cognitive impairment, and Alzheimer's diease. Biomed Res Int. (2005) 2015:748212 10.1155/2015/748212 PMC4624908 26550575

[7] Law LLF Barnett F Yau MK Gray MA. Effects of combined cognitive and exercise interventions on cognition in older adults with and without cognitive impairment: a systematic review. Ageing Res Rev. (2014) 15:61–75. 10.1016/j.arr.2014.02.008 24632497

[8] Cheng ST Chow PK Song YQ Yu EC Lam JH. Can leisure activities slow dementia progression in nursing home residents? a cluster-randomized controlled trial. Int Psychogeriatr. (2014) 26:637–43. 10.1017/S1041610213002524 24411480

[9] Man LC Chang MY Chu MC Effects of mahjong on the cognitive function of middle-aged and older people. Int J Geriatr Psychiatry. (2015) 30:994–7. 10.1002/gps.4307 26220879

[10] Cacciaglia R Molinuevo JL Sanchez-Benavides G Falcón C Gramunt N Brugulat-Serrat A. Episodic memory and executive functions in cognitively healthy individuals display distinct neuroanatomical correlates which are differentially modulated by aging. Hum Brain Mapp. (2018) 39:4565–79. 10.1002/hbm.24306 29972619 PMC6220988

[11] Tsang WWN Wong GCK Gao KL Mahjong playing and eye-hand coordination in older-a cross-sectional study. J Phys Ther Sci. (2016) 28:2955–60. 10.1589/jpts.28.2955 27821969 PMC5088160

[12] Ginsberg TB Powell L Emrani S Wasserman V Higgins S Chopra A. Instrumental activities of daily living, neuropsychiatric symptoms, and neuropsychological impairment in mild cognitive impairment. J Am Osteopath Assoc. (2019) 119:96–101. 10.7556/jaoa.2019.015 30688355

[13] Knight MJ Baune BT. Executive function and spatial cognition mediate psychosocial dysfunction in major depressive disorder. Front psychiatry. (2018) 9:539. 10.3389/fpsyt.2018.00539 30420817 PMC6215806

[14] Boey KW Chiu HFK Assessing psychological well-being of the old-old: a comparative study of GDS-15 and GHQ-12. Clin Gerontol. (1998) 19:65–75. 10.1300/J018v19n01_06

[15] Boey KW The use of GDS-15 among the older adults in Beijing. Clin Gerontol. (2000) 21:49–60. 10.1300/J018v21n02_05

[16] de Oliveira Silva F Ferreira JV Plácido J Sant'Anna P Araújo J Marinho V Three months of multimodal training contributes to mobility and executive function in elderly individuals with mild cognitive impairment, but not in those with Alzheimer's disease: a randomized controlled trial. Maturitas. (2019) 126:28–33. 10.1016/j.maturitas.2019.04.217 31239114

[17] Liao YY Chen IH Lin YJ Chen Y Hsu WC. Effects of virtual reality-based physical and cognitive training on executive function and dual-task gait performance in older adults with mild cognitive impairment: a randomized control trial. Front Aging Neurosci. (2019) 11:162. 10.3389/fnagi.2019.00162 31379553 PMC6646677

[18] Cheng ST Chan ACM Yu ECS. An exploratory study of the effect of mahjong on the cognitive functioning of persons with dementia. Int J Geriatr psychiatry. (2006) 21:611–7. 10.1002/gps.1531 16779765

[19] Yu J Li J Huang X The Beijing version of the montreal cognitive assessment as a brief screening tool for mild cogvitive impairment: a community-based study. BMC Psychiatry. (2012) 25:156 10.1186/1471-244X-12-156 PMC3499377 23009126

[20] Zhao Q Guo Q Li F Zhou Y Wang B Hong Z. The shape trail test: application of a new variant of the trail making test. PLoS ONE. (2013). 8:e57333. 10.1371/journal.pone.0057333 23437370 PMC3577727

[21] Pfeffer RI Kurosaki TT Harrah CH Jr Chance JM Filos S. Measurement of functional activities in older adults in the community. J Gerontol. (1982) 37:323–9. 10.1093/geronj/37.3.323 7069156

[22] Nasreddine ZS Phillips NA Bédirian V Charbonneau S Whitehead V Collin I. The montreal cognitive assessment, MoCA: a brief screening tool for mild cognitive impairment. Am Geriatr Soc. (2005) 53:695–9. 10.1111/j.1532-5415.2005.53221.x 15817019

[23] Wen HB Zhang ZX Niu FS Li L. [The application of montreal cognitive assessment in urban Chinese residents of Beijing]. Chin J Intern Med. (2008) 47:36–39. 10.3969/j.issn.1006-2963.2014.04.006 18346324

[24] Lu JC Guo QH Hong Z Trail making test used by Chinese elderly patients with mild cognitive impairment and mild Alzheimer dementia. Chin J Clin Psychol. (2006) 14:118–21. 10.3969/j.issn.1005-3611.2006.02.003

[25] Li X Jia S Zhou Z Jin Y Zhang X Hou C. The role of the Montreal Cognitive Assessment (MoCA) and its memory tasks for detecting mild cognitive impairment. Neurol Sci. (2018) 39:1029–34. 10.1007/s10072-018-3319-0 29550982

[26] Zhang Xueqing ZH Cut-off value of montreal cognitive assessment: a layering research in screening of elderly with mild cognitive impairment in ChangSha city. Chin General Pract. (2014) 17:3046–3051. 10.3969/j.issn.1007-9572.2014.26.003

[27] Arbuthnott K Frank J. Trail making test, part B as a measure of executive control: validation using a set-switching paradigm. J Clin Exp Neuropsychol. (2010) 22:518–28. 10.1076/1380-3395(200008)22:4;1-0;FT518 10923061

[28] Nitrini R Caramelli P Herrera E Jr Bahia VS Caixeta LF Radanovic M. Incidence of dementia in a community-dwelling Brazilian population. Alzheimer Dis Assoc Disord. (2004) 18:241–6. 15592138

[29] Bezdicek O Stepankova H Martinec Novakova L Kopecek M. Toward the processing speed theory of activities of daily living in healthy aging: normative data of the functional activities questionnaire. Aging Clin Exp Res. (2016) 28:239–47. 10.1007/s40520-015-0413-5 26231091

[30] Guarino A Favieri F Boncompagni I Agostini F Cantone M Casagrande M. Executive functions in Alzheimer disease: a systematic review. Front Aging Neurosci. (2018) 10:437. 10.3389/fnagi.2018.00437 30697157 PMC6341024

[31] Garcia-Alvarez L Gomar JJ Sousa A Garcia-Portilla MP Goldberg TE. Breadth and depth of working memory and executive function compromises in mild cognitive impairment and their relationships to frontal lobe morphometry and functional competence. Alzheimers Dement (Amst). (2019) 11:170–9. 10.1016/j.dadm.2018.12.010 30911598 PMC6416209

[32] Kim GH Jeon S Im K Kwon H Lee BH Kim GY. Structural brain changes after traditional and robot-assisted multi-domain cognitive training in community-dwelling healthy elderly. PLoS ONE. (2015) 10:e0123251. 10.1371/journal.pone.0123251 25898367 PMC4405358

[33] Lövdén M Bodammer NC Kühn S Kaufmann J Schütze H Tempelmann C. Experience-dependent plasticity of white-matter microstructure extends into old age. Neuropsychologia. (2010) 48:3878–83. 10.1016/j.neuropsychologia.2010.08.026 20816877

[34] Chapman SB Aslan S Spence JS Hart JJ Jr Bartz EK Didehbani N. Neural mechanisms of brain plasticity with complex cognitive training in healthy seniors. Cereb Cortex. (2015) 25:396–405. 10.1093/cercor/bht234 23985135 PMC4351428

[35] Cao W Cao X Hou C Li T Cheng Y Jiang L. Effects of cognitive training on resting-state functional connectivity of default mode, salience, and central executive networks. Front Aging Neurosci. (2016) 8:70. 10.3389/fnagi.2016.00070 27148042 PMC4828428

[36] Zheng WY Walker M Blaszczynski A. Mahjong gambling in the Chinese-australian community in Sydney: a prevalence study. J Gambl Stud. (2010) 26:441–54. 10.1007/s10899-009-9159-3 19936893

[37] Yang Li XL Comparative of four neuropsychological scales in early diagnosis of Alzheimer's disease. J Chongqing Med Univ. (2014) 39:488–92. 10.13406/j.cnki.cyxb.000085

[38] Mazzeo S Padiglioni S Bagnoli S Bracco L Nacmias B Sorbi S Bessi V. The dual role of cognitive reserve in subjective cognitive decline and mild cognitive impairment: a 7-year follow-up study. J Neurol. (2019) 266:487–97. 10.1007/s00415-018-9164-5 30604054

[39] Ratiu I Virden TB Baylow H Flint M Esfandiarei M. Executive function and quality of life in individuals with marfan syndrome. Qual Life Res. (2018) 27:2057–65. 10.1007/s11136-018-1859-7 29671248

[40] Rog LA Park LQ Harvey DJ Huang CJ Mackin S Farias ST. The independent contributions of cognitive impairment and neuropsychiatric symptoms to everyday function in older adults. Clin Neuropsychol. (2014) 28:215–36. 10.1080/13854046.2013.876101 24502686 PMC4021718

[41] Giles GM Clark-Wilson J Baxter DM Tasker R Holloway M Seymour S. The interrelationship of functional skills in individuals living in the community, following moderate to severe traumatic brain injury. Brain Inj. (2019) 33:129–36. 10.1080/02699052.2018.1539762 30424682

[42] Kumar KS Samuelkamaleshkumar S Viswanathan A Macaden AS. Cognitive rehabilitation for adults with traumatic brain injury to improve occupational outcomes. Cochrane Database Syst Rev. (2017) 6:CD007935. 10.1002/14651858.CD007935.pub2 28631816 PMC6481568

[43] Tang F Zhang Dong. Activity engagement and cognitive function: findings from a community-dwelling U.S. Chinese aging population study. Gerontol Geriatr Med. (2018) 4:2333721418778180. 10.1177/2333721418778180 30035190 PMC6050630

[44] Bourassa KJ Memel M Woolverton C Sbarra DA. Social participation predicts cognitive functioninging aging adults over time: Comparisons with physical health, depression, and physical activity. Aging Ment Health. (2017) 21:133–46. 10.1080/13607863.2015.1081152 26327492

[45] Gao M Sa Z Li Y Zhang W Tian D Zhang S. Does social participation reduce the risk of functional disability among older adults in China? a survival analysis using the 2005–2011 waves of the CLHLS data. BMC Geriatr. (2018) 18:224. 10.1186/s12877-018-0903-3 30241507 PMC6151053

[46] Kobayashi-Cuya KE Sakurai R Sakuma N Suzuki H Yasunaga M Ogawa S Hand dexterity, not handgrip strength, is associated with executive function in Japanese community-dwelling older adults:a cross-sectional study. BMC Geriatr. (2018) 18:192 10.1186/s12877-018-0880-6 30143006 PMC6109297

[47] Ige JGL Bray I Gray S. Methods of identifying and recruiting older people at risk of social isolation and loneliness: a mixed methods review. BMC Med Res Methodol. (2019) 19:181. 10.1186/s12874-019-0825-6 31464586 PMC6714404

[48] Mikkelsen ASB Petersen S Dragsted AC Kristiansen M. Social interventions targeting social relations among older people at nursing homes: a qualitative synthesized systematic review. Inquiry. (2019) 56:46958018823929. 10.1177/0046958018823929 30791836 PMC6376508

[49] Teh JKL Tey NP. Effects of selected leisure activities on preventing loneliness among older Chinese. SSM Population Health. (2019) 9:100479. 10.1016/j.ssmph.2019.100479 31646167 PMC6804430

[50] Subramaniam M Abdin E Shahwan S Vaingankar JA Picco L Browning CJ. Culture and age influences upon gambling and problem gambling. Addict Behav Rep. (2015) 1:57–63. 10.1016/j.abrep.2015.04.004 29531980 PMC5845977

[51] Fukuma K Ihara M Miyashita K Fukuma K Ihara M Miyashita K. Right parietal source in mahjong-induced seizure: a system epilepsy of focal origin. Clin Case Rep. (2016) 4:948–51. 10.1002/ccr3.653 27761244 PMC5054468

